# Adverse Childhood Experiences, Personality, and Crime: Distinct Associations among a High-Risk Sample of Institutionalized Youth

**DOI:** 10.3390/ijerph19031227

**Published:** 2022-01-22

**Authors:** Steffen Barra, Marcel Aebi, Delfine d’Huart, Klaus Schmeck, Marc Schmid, Cyril Boonmann

**Affiliations:** 1 Institute for Forensic Psychology and Psychiatry, Saarland University Hospital, Kirrberger Str. 100, 66421 Homburg, Germany; 2 Research & Development, Corrections and Rehabilitation, Department of Justice and Home Affairs, Hohlstrasse 552, 8090 Zurich, Switzerland; marcel.aebi@uzh.ch; 3 Child and Adolescent Psychiatry, Medical Faculty, University of Zurich, Neumuensterallee 3, 8032 Zurich, Switzerland; 4 Child and Adolescent Psychiatric Research Department, Psychiatric University Hospitals, University of Basel, Wilhelm Klein-Strasse 27, 4002 Basel, Switzerland; Delfine.d'Huart@upk.ch (D.d.); klaus.schmeck@unibas.ch (K.S.); marc.schmid@upk.ch (M.S.); cyril.boonmann@upk.ch (C.B.); 5 Department of Forensic Child and Adolescent Psychiatry, Psychiatric University Hospitals, University of Basel, Wilhelm Klein-Strasse 27, 4002 Basel, Switzerland

**Keywords:** adverse childhood experiences, trauma, personality, psychopathy, temperament, personality disorder, psychopathology, delinquency, reoffending, child welfare, residential care

## Abstract

Despite high rates of adverse childhood experiences (ACEs) and personality-related disturbances among delinquent juveniles, associations among ACEs, youth personality, and juvenile crime involvement are still unclear. High-risk samples of institutionalized youth are in specific need of a comprehensive assessment of ACEs and personality features in order to broaden the current knowledge on the occurrence and persistence of juvenile crime and to derive implications for prevention and intervention. We examined a heterogeneous high-risk sample of 342 adolescents (35.1% females, 64.9% males) aged between 12 and 18 years (*M* = 15.74, *SD* = 1.61 years) living in child-welfare or juvenile justice institutions regarding cumulative ACEs, psychopathic traits, temperament, and clinical personality disorder ratings, and criminal involvement before and up to 10 years after assessment. We found considerable rates of ACEs, although cumulative ACEs did not predict future crime. Latent Profile Analysis based on dimensional measures of psychopathy, temperament, and personality disorders derived six distinct personality profiles, which were differently related to ACEs, personality disturbances, clinical psychopathology, and future delinquency. A socially difficult personality profile was associated with increased risk of future crime, whereas avoidant personality traits appeared protective. Findings indicate that the role of ACEs in the prediction of juvenile delinquency is still not sufficiently clear and that relying on single personality traits alone is insufficient in the explanation of juvenile crime.

## 1. Introduction

Adolescence displays a developmental period in which delinquent behaviors are most common, with some young people showing persistent crime involvement until adulthood [1]. Prior studies have investigated a wide variety of influencing factors in order to explain what contributes to young people’s first and repeated criminal conduct. Despite situational factors that enhance a juvenile’s risk to commit criminal behaviors, research has claimed that juvenile crime may be partly explainable by psychosocial burden including adverse childhood experiences (ACEs) as well as maladaptive personality development, especially in those youth who show continuous criminal careers.

ACEs are common among young offenders and have been linked to increased risk of (repeated) juvenile crime involvement, especially when several ACEs occur in an cumulative manner [2,3,4,5,6]. Several theoretical models have been proposed to explain the associations of ACEs with criminal behavior, e.g., the General Aggression Model (GAM) [7] or General Strain Theory [8], which include both external (social) and internal (emotional and cognitive) processes. However, neither theory may yet sufficiently explain the ACE-delinquency link. Although GAM, for example, also stressed that an aggressive personality increases the risk of perpetrating aggressive behavior, most theoretical models about ACEs and crime have not yet included individual personality features. However, increasing rates of ACEs were found to influence maladaptive personality development [9,10,11,12,13], and certain aspects of adolescent personality appear to be associated with an elevated risk of crime perpetration. As such, psychopathic traits have often been investigated in the context of criminal behavior. The construct of psychopathy consisting of affective (e.g., lack of guilt and empathy, shallow affect), interpersonal (e.g., grandiosity and manipulativeness), and behavioral (e.g., impulsivity and irresponsibility) personality features was found important to assess in seriously offending adults [14]. Furthermore, Salekin and Frick [15] highlighted the development of psychopathic features in children and adolescents to explain behavioral problems. Coles et al. [16] reported elevated psychopathic and paranoid traits in incarcerated male adolescents between the ages of 13–18 years. DeLisi et al. [17] found that psychopathic traits were related to criminal onset in young delinquents. In their meta-analysis of 53 studies, Asscher et al. [18] concluded that (recidivistic) criminal activities could be predicted by psychopathy within the transitional period between middle childhood and adolescence and, thus, that psychopathic traits should be screened for as early as possible to prevent (continuous) crime involvement. 

Although a vast number of studies have focused on psychopathy as highly relevant personality feature in the context of crime, others have pointed to the need to evaluate further personality characteristics, too. As such, temperament has recently gained more scientific attention as a potentially relevant personality construct related to juvenile crime. In their temperament-based theory of crime, DeLisi and Vaughn [19] define temperament as the individual ability to regulate emotions and behaviors, especially when interacting with others. They state that conduct problems or maladaptive social (criminal) behavior may result from deficient self-regulation skills and negative emotionality. Ljubin-Golub et al. [20] underlined the role of temperament in terms of sensation seeking for rather minor juvenile delinquency, but not violent crime. 

Furthermore, Tackett et al. [11] point to an increased risk of future violence and crime related to the Diagnostic and Statistical Manual of Mental Disorders’ (DSM) Cluster A and B, but not Cluster C personality disorders in adolescence. Sevecke et al. [21] found that—compared to clinically referred adolescents, delinquent juveniles showed higher rates of DSM Cluster B personality disorders, especially narcissistic and antisocial personality disorders. Whereas paranoid, narcissistic, and antisocial personality disorders were most prevalent among young male offenders, females reported higher rates of borderline personality disorder. Krischer et al. [22] did also find higher rates of personality disorders, but also elevated scores on psychopathic traits, in criminal compared to non-criminal youth. They discussed a specific criminal personality profile characterized by dissocial behaviors with particularly high scores on conduct problems and stimulus seeking. The authors further concluded that delinquency was related to affective lability, callousness, and impulsivity and, thus, recommended to consider both general (non-pathological) and maladaptive (pathological) personality features.

The associations among ACEs, youth personality, and juvenile crime involvement are yet unclear. Farina et al. [23] detected significant predictive effects of increasing degrees of ACEs on psychopathic traits among male and female institutionalized juvenile offenders. Examining a sample of detained and non-detained adolescent males and females, Krischer and Sevecke [24] found associations between physical and emotional ACEs and psychopathy in detained boys, whereas findings for girls were inconclusive. Perez et al. [25] stated that cumulative ACEs were associated with severe and chronic crime in adolescents, but that this association was mediated by maladaptive personality traits such as impulsivity and aggression. Implementing path analysis on data of male adolescents, DeLisi et al. [26] concluded that psychopathy partially mediated associations between ACEs and juvenile delinquency and fully mediated associations between ACEs and proactive overt aggression. Moreover, testing the abovementioned temperament-based theory of crime by DeLisi and Vaughn [19], DeLisi et al. [27] found that temperament was more strongly associated with delinquency than ACEs and psychopathic traits in juvenile offenders.

Overall, research on the role of ACEs and personality traits in the development and maintenance of juvenile criminal behaviors is limited, and existing findings are rather inconclusive. Most studies rely on either small or only justice-involved samples, which impedes the generalizability of implications to adolescents who have not yet engaged in criminal behaviors, although respective knowledge would be highly valuable for early crime prevention. Especially, adolescents in child welfare institutions or residential placement can be described as high-risk samples in the context of ACEs, personality-related and psychopathological disturbances, and criminal conduct. Garcia et al. [28], for example, reported high rates of ACEs among young people within the child welfare system. Zettler et al. [29] found that ACEs increased the risk of juvenile residential placement. In their systemic review, Kvamme et al. [30] emphasized high rates of ACEs, clinical psychopathology, and future delinquency among young people leaving residential placement (mostly forensic institutions). Baglivio et al. [31] stated that cumulative ACEs had no direct effects on criminal recidivism in delinquent adolescents placed in juvenile justice residential care, but were indirectly associated with future crime through child welfare involvement. Although a number of studies addressed ACEs in child welfare settings (e.g., [28,32]), it remains unclear how ACEs and personality features relate to future criminal behaviors in institutionalized youth.

Furthermore, studies have often examined single types of ACEs (e.g., physical and emotional abuse) and single personality traits (e.g., psychopathy, temperament, or personality disorders) separately instead of taking their co-existence into account by implementing a more holistic approach to examine the question of whether and how different ACEs and aspects of personality (both non-pathological and pathological) influence juvenile crime involvement, either distinctively or in combination. In recent years, research has tried to take the co-occurrence of ACEs among high-risk youth samples into account not only by creating cumulative ACE scores, but also by implementing person-centered approaches such as Latent Class Analysis (LCA) (e.g., [4,33]). Others have investigated patterns of criminal behaviors by LCA or profiles of psychiatric disturbances among delinquent youth by Latent Profile Analysis (LPA) and examined their relations to cumulative ACEs [34,35]. Moreover, a growing number of studies has analyzed specific personality profiles using LPA in samples of justice-involved youth based on rather general personality traits [36] or psychopathy [37]. However, to the best of our knowledge, no study has yet considered both non-pathological and pathological personality traits simultaneously in order to empirically derive specific personality profiles among high-risk youth samples. By the simultaneous use of various personality measures, specific adaptive and maladaptive personality profiles may be assessed in adolescent high-risk samples. Hicks and colleagues [38] discuss the advantages and shortcoming of both person-centered and variable-centered approaches in the examination of personality traits, whereas variable-centered approaches are considered useful to describe single personality constructs and their associations with other constructs or outcomes across or between individuals. Person-centered approaches allow for the consideration of the co-existence and interdependence of different personality constructs within an individual. Thus, they not only serve to disentangle the heterogeneity of personality traits among populations by empirically deriving homogeneous subgroups based on specific personality patterns, but they are also beneficial to gain a more sophisticated knowledge on etiological and phenotypic features of personality and their associations to certain outcomes over and above the reliance on single dimensional traits. Hicks at al. stress that both approaches should be considered complementary to gain a deeper understanding of human personality [38]. 

Moreover, previous studies have mostly examined cross-sectional associations between ACEs, personality, and crime (e.g., by comparing delinquent with non-delinquent samples) instead of longitudinal relations on how ACEs and personality may predict future delinquency in high-risk samples of both delinquent and non-delinquent adolescents. The latter is, however, of specific importance to inspire early treatment and prevent the occurrence and continuation of young people’s criminal behaviors. 

Thus, we implemented a more comprehensive approach in the present study to answer the question of whether and how ACEs and different aspects of youth personality (both non-pathological and pathological in terms of psychopathic traits, temperament, and personality disorders) influence future crime involvement in a heterogeneous high-risk sample of institutionalized male and female adolescents placed under civil and penal law, or voluntarily, with and without prior criminal conduct. 

We aimed at considering a broad, integrative conceptualization of youth personality by implementing the person-centered approach of LPA to empirically derive personality profiles based on dimensional measures of psychopathy, temperament, and DSM personality disorders. Furthermore, we followed the claim by Hicks and colleagues [38] and additionally conducted variable-centered analyses. With respect to previous findings suggesting that ACEs seldomly appear in isolation (especially in high-risk youth samples), we included a cumulative ACE score. Based on previous research, we expected to find (1) high rates of ACEs and psychopathological burden among a high-risk sample of institutionalized youth, (2) distinct personality profiles with at least one highly disturbed profile and one rather inconspicuous profile, and (3) higher rates of ACEs in highly disturbed personality profiles. Due to the ambiguity of research related to the predictive effects of ACEs and personality features on future criminal behavior, we examined respective associations in an exploratory manner. In addition to personality profiles, we further tested dimensional measures of personality as predictors of future crime involvement.

## 2. Materials and Methods

### 2.1. Study Design

Data was obtained from the longitudinal “Swiss Study for Clarification and Goal-Attainment in Child Welfare and Juvenile-Justice Institutions” (German: Modellversuch Abklärung und Zielerreichung in stationären Massnahmen; MAZ., [39]). The MAZ. study was conducted between 2007 and 2012 with the primary aim of describing mental health and offending behavior of children and adolescents in child welfare and residential care/juvenile justice institutions. Respective institutions accredited by the Swiss Federal Ministry of Justice were invited to participate, of which 64 institutions (35%) agreed to take part. These 64 institutions served as representation for the different types of Swiss youth institutions, e.g., regarding size, schooling, treatment options, and residing children and adolescents (see also [40]). Juveniles who had been living for at least 1 month in one of these 64 institutions with sufficient language skills in German, French, or Italian as well as sufficient intelligence scores (IQ > 70) were eligible for participation. Prior to participation, the juveniles as well as their legal guardians and social caseworkers received oral and written information about the study and were asked to give their informed consent. Participants then completed computer-assisted self-report questionnaires regarding mental health, psychosocial problems, ACEs, and personality traits. In addition, a social caseworker was selected for each participant to answer similar questionnaires related to that participant. The selected caseworkers were required to know the participant for at least 1 month and to confirm that they felt confident to validly answer the questionnaires. Additionally, participants were assessed for mental and personality disorders as well as ACEs using semi-structured clinical interviews. The assessment was conducted by trained psychologists and research assistants. The study procedure was approved by the Ethics Committees on Research Involving Humans at the University of Basel and the University of Lausanne (Switzerland) and by the Institutional Review Board at the University of Ulm (Germany).

### 2.2. Participants

Overall, 592 children and adolescents aged 6–26 years (*M* = 16.3 years) participated in the MAZ. study at baseline. As the primary aim of the current study was to investigate distinct personality profiles based on psychopathic traits, temperament, and measures of DSM personality disorders, and their associations with ACEs and (future) crime involvement, only participants with complete information on the below-mentioned assessment instruments for ACEs and personality traits were included in the present analyses. Taking into account the age limits for usage of some of these instruments (i.e., YPI, JTCI), participants younger than 12 years or older than 18 years were excluded. Data on crime involvement was available for all these participants. The final sample included 342 participants (35.1% female) with a mean age of 15.74 years (*SD* = 1.61, range = 12–18). Most of them were of Swiss nationality (85.7%), and 23.2% came from families with low socio-economic status (SES). Most of the participants (58.2%) were placed under civil law, whereas 17.3% were placed under penal law, and 18.1% were placed voluntarily. Female and male participants did not differ concerning age, nationality, or SES. However, differences emerged regarding the reasons for placement, with proportionally more female participants being placed under civil law (female: 72.6%, male: 52.5%, adjusted residuals (*AR*) = 3.6) and more male participants being placed under penal law (female: 6.8%, male: 23.5%, *AR* = 3.8), χ^2^ (5) = 20.07, *p* ≤ 0.001.) Excluded participants were somewhat older than included participants (*M* = 16.58 years, *SD* = 3.60), *T* (514) = 6.89, *p* ≤ 0.001, were more often placed under penal law (36.0%, *AR* = 5.0), and less often placed under civil law (45.7%, *AR* = −3.3) than included participants, χ^2^ (5) = 28.86, *p* ≤ 0.001. Distributions of sex, χ^2^ (1) = 3.33, *p* = 0.068, nationality, χ^2^ (1) = 0.68, *p* = 0.410, or low SES, χ^2^ (1) = 0.14, *p* = 0.705 did not differ.

### 2.3. Measurements

#### 2.3.1. ACEs

ACEs were assessed using the Essen Trauma-Inventory for Children and Adolescents (ETI-CA; [41]). Participants were given a list of 15 potentially traumatic experiences (i.e., natural disaster; severe accident, fire or explosion; severe illness; violent assault by stranger; violent assault by family member/acquaintance; death of a caregiver; imprisonment; sexual abuse by stranger (before age 18); sexual abuse by stranger (since age 18); sexual abuse by family member/acquaintance (before age 18); sexual abuse by family member/acquaintance (since age 18); war experience; torture; emotional/physical neglect; others), and were asked if they had ever experienced any of these situations personally, as a witness, or both. We used a cumulative ACE measure (ETI sum score) for the current study. The ETI-CA developers reported good to very good reliability scores (Cronbach’s α = 0.80–0.94; [41]). In the present sample, the ETI sum score showed acceptable internal consistency (Cronbach’s α = 0.70).

#### 2.3.2. Psychopathic Traits

Psychopathic traits were assessed with the Youth Psychopathic Traits Inventory (YPI; [42]). The YPI is a 50-item self-report questionnaire for adolescents aged 12–18 years, designed to assess psychopathic traits on 10 subscales combined into three core dimensions, namely the grandiose-manipulative dimension (i.e., dishonest charm, grandiosity, lying, and manipulation subscales), the callous-unemotional dimension (i.e., callousness, lack of emotion, and remorselessness subscales), as well as the impulsive-irresponsible dimension (i.e., impulsivity, thrill-seeking, and irresponsibility subscales). Each item is rated on a 4-point Likert scale (1 = does not apply at all, 2 = does not apply well, 3 = applies fairly well, 4 = applies very well), with higher scores reflecting increased levels of psychopathic traits. Internal consistency of the YPI subscales was proven to be good to excellent, (Cronbach’s α = 0.66–0.93; [42]). In the present sample, the YPI sum score showed excellent internal consistency (Cronbach’s α = 0.92).

#### 2.3.3. Temperament

Temperament was assessed using the JTCI-12-18-R [43]. The JTCI is a self-administered questionnaire for children and adolescents aged 12–18 years based on the biopsychosocial model by Cloninger [44]. The four temperament scales included in this study measure (1) novelty seeking (explorative behavior, impulsive decision making, speed and intensity of an emotional reaction, active avoidance of frustration, and tendencies to exceed rules in the course of it), (2) harm avoidance (passive-avoidant tendencies such as anxiety, shyness, pessimistic worries, and fatigue), (3) reward dependence (spontaneous sensitivity and warmth as well as maintaining stable social relationships), and (4) persistence (readiness for hard work, ambition, perseverance, and perfectionism). All scales were based on cumulative sum scores of 13–18 items that were rated on a five-point rating scale (0 = not true to 4 = very true). The retest reliability was about 0.68 and internal consistency varied between 0.79 and 0.85 in the original validation sample [43]. In the present sample, internal consistency ranged from Cronbach’s α = 0.74–0.80.

#### 2.3.4. Personality Disorder Traits

Personality disorder (PD) traits were assessed with the Structured Clinical Interview for DSM-IV-TR Axis II Personality Disorders (SCID-II; e.g., [45]). The SCID-II is a semi-structured interview designed to yield PD diagnoses based on the DSM-IV and DSM-IV-TR (i.e., paranoid, schizoid, schizotypal, histrionic, borderline, antisocial, narcissistic, avoidant, dependent, obsessive-compulsive, depressive, passive-aggressive PDs). First, a screening questionnaire was administered by the participants with 134 items, which are rated on a 3-point Likert scale (1 = absent, 2 = subthreshold, 3 = threshold). Dimensional scores are provided by summing the scores from each individual item for each separate PD category. Second, categorical diagnoses were provided according to the specific diagnostic thresholds of PDs by trained clinicians. Interrater reliability for dimensional diagnoses varies from 0.90 to 0.98 (interclass correlation), and internal consistency (Cronbach’s α) ranges from 0.71 to 0.94 [45]. For the present study, we used self-rated, dimensional PD traits for main analyses and categorical, clinician-administered PD diagnoses for descriptive purpose. We combined single PD categories into DSM-Clusters: Cluster A (paranoid, schizoid, schizotypal; Cronbach’s α = 0.93), Cluster B (histrionic, borderline, antisocial, narcissistic; Cronbach’s α = 0.93), Cluster C (avoidant, dependent, obsessive-compulsive; Cronbach’s α = 0.89), and others (depressive, passive-aggressive; Cronbach’s α = 0.87). 

#### 2.3.5. Psychiatric Disorders

Psychiatric disorders were assessed with the Schedule for Affective Disorders and Schizophrenia for School-Age Children—Present and Lifetime Version (K-SADS-PL; [46]). The K-SADS-PL is a semi-structured clinical interview that provides a reliable and valid measurement of current and lifetime DSM-IV diagnoses (i.e., affective disorders, anxiety disorders, psychotic disorders, behavioral disorders, substance abuse, eating disorders, and tic disorders) in children and adolescents aged 6–18 years. Individual responses are rated on a 4-point Likert scale (0 = no information available, 1 = absent, 2 = subthreshold, 3 = threshold). Interrater agreement in scoring screens and diagnoses is high, and test-retest reliability (Cohen’s κ) ranges between 0.77 and 1.00 for current and/or lifetime diagnoses of major depression, any bipolar, generalized anxiety, conduct, and oppositional defiant disorders, as well as between 0.63 and 0.67 for current diagnoses of posttraumatic stress disorder (PTSD) and attention-deficit hyperactivity disorder (ADHD) [46]. 

#### 2.3.6. Delinquency

Data on participants’ officially recorded criminal convictions was obtained from the Swiss Federal Ministry of Statistics until the end of 2017, up to 10 years after the initial assessment of the study (observation period of 6–10 years after assessment, *M* = 8.47 years, *SD* = 1.10 years). For the present study, we included both convictions before (prior delinquency) and after assessment (future delinquency) with regard to the following categories: any delinquency, violent delinquency (e.g., bodily harm/mayhem, homicides), and non-violent delinquency (e.g., theft, drug related crime).

#### 2.3.7. Sociodemographic Characteristics

Sociodemographic information on age, sex, nationality, and SES was collected using a computer-based questionnaire. Youth whose parents both (or one in case of missing information on the other) were out of work or unskilled workers as categorized by the International Standard Classification of Occupations (ISCO-08) guidelines [47] were considered to come from families with low SES.

### 2.4. Statistical Analyses

Data were analyzed in IBM SPSS version 28.0 (IBM Corp, Armonk, NY, USA) for Windows and in R [48]. We conducted LPA using the tidyLPA package in R [49] to empirically derive youth personality profiles based on z-transformed YPI sum scores, JTCI temperament scales’ sum scores (novelty seeking, harm avoidance, reward dependence, and persistence) and sum scores on the four DSM personality disorder Clusters A, B, C, and others assessed by SCID-II self-ratings. Models with one to nine latent profiles were compared regarding best data fit. Balance of model fit and parsimony increases with decreasing fit indices. Several fit indices were considered to identify the best fitting model. First, the Akaike Information Criterion [50] and the Bayesian Information Criterion [51] were considered. Second, we applied a hierarchical analytical process provided by the tidyLPA command in R [52] that additionally considered the Approximate Weight of Evidence Criterion [53], Classification Likelihood Criterion [54], and Kullback Information Criterion [55]. We also conducted Bootstrapped parametric Likelihood Ratio Tests (BLRT; [56]) with significant results indicating that a k-class model fits the data better than a (k-1)-class model. Individual assignment to latent profiles was conducted under consideration of the highest affiliation probability based on maximum likelihood estimations. Differences among distinct personality profiles regarding ACEs, psychiatric disorders, prior delinquency, and sociodemographic characteristics were examined by parametric and non-parametric analyses, e.g., χ^2^-statistics, ANOVAs, and MANOVAs with post-hoc Bonferroni or Games–Howell tests. Predictive associations of ACEs and personality profiles/traits with future delinquency were tested by univariate and multivariate binary logistic regression analyses. The global level of significance was set to be at least *p* ≤ 0.05.

## 3. Results

### 3.1. Prevalence of ACEs, Personality Traits, Psychopathology, and Crime

Appendix A (Table A1) displays the distribution of the abovementioned variables of interest in the total sample. More than 82% of the sample reported at least one ACE, and more than half of the juveniles had experienced at least three different ACEs. Clinician-administered personality disorders were present in more than 20% of the sample, with half of them showing combined/unspecified personality disorders followed by Cluster B disorders. More than 82% of the sample showed at least one psychiatric disorder (based on K-SADS-PL), about one quarter of the sample at last three. Among psychiatric disorders, conduct disorders were most commonly reported, followed by affective disorders and ADHD. About one third of the sample had been convicted for any criminal behavior before as well as after the assessment. Non-violent offenses were reported more frequently than violent offenses (cumulative percentages exceed 100 as some youth showed both violent and non-violent offending).

### 3.2. LPA on Dimensional Personality Traits

**Figure 1:**
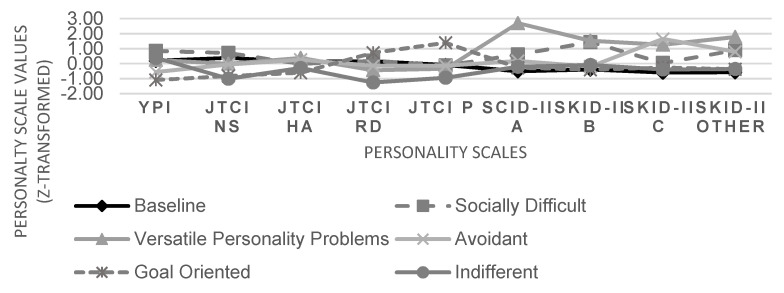
LPA six-profile model based on YPI, JTCI temperament scales, and dimensional SCID-II cluster ratings (z-transformed).

Table 1 displays the results of model comparisons based on AIC, BIC, and BLRT. The AIC favored the eight-profile solution, the BIC pointed to the six-profile solution. The BLRT indicated that gradually increasing profile numbers were associated with better data fit until the eight-profile model, whereas a nine-profile solution did not significantly fit the data better than an eight-profile model. Finally, the hierarchical analytical process provided by the tidyLPA command in R [52] favored a six-profile model. In addition, the six derived personality profiles were easily interpretable. Individual assignment of participants to latent profiles was sufficiently clear (entropy = 0.82; [57]). Thus, we chose the six-profile model for further analyses. Figure 1 displays the six distinct profiles (based on standardized z-values) that we labeled (1) baseline (*n* = 144, 42.1%), (2) socially difficult (highest YPI sum score, high on novelty seeking and SCID-II Cluster B scores; *n* = 37, 10.8%), (3) versatile personality problems (high on each SCID-II scale; *n* = 23, 6.7%), (4) avoidant (high on SCID-II Cluster C scores; *n* = 50, 14.6%), (5) goal oriented (low on clinical personality problems, high on reward dependence and persistence; *n* = 49, 14.3%), and (6) indifferent (low on clinical personality problems; low on novelty seeking, reward dependence, and persistence; *n* = 39, 11.4%).

**Table 1:** Model parameters of latent profile analyses based on psychopathy, temperament, and personality disorder ratings.(*N* = 342).

Model	AIC	BIC	BLRT (p)	Entropy
1 Class	21,993.40	22,062.42	-	1.00
2 Class	21,593.79	21,701.17	0.01	0.90
3 Class	21,518.13	21,663.85	0.01	0.77
4 Class	21,424.73	21,668.80	0.01	0.74
5 Class	21,343.48	21,565.89	0.01	0.79
6 Class	21,235.22	21,495.99	0.01	0.82
7 Class	21,227.77	21,526.89	0.02	0.79
8 Class	21,203.84	21,541.30	0.01	0.82
9 Class	21,216.64	21,532.45	0.91	0.76

Note. AIC = Akaike Information Criterion; BIC = Bayesian Information Criterion; BLRT = Bootstrapped Likelihood Ratio Test.

### 3.3. Differences among LPA Personality Profiles

Table 2 displays the distribution of the main variables of interest in the six distinct personality profiles (for more details, see Appendix A, Table A1). Although no differences among LPA profiles emerged concerning cumulative ACEs (ETI sum score), youth from the baseline profile were more likely to have experienced no ACEs, and youth from the baseline and the goal-oriented profiles were less likely to have experienced more than three ACEs compared to the other profiles. Youth from the versatile personality problems profile were most likely to have experienced more than three ACEs. Clinician-administered personality disorders were more likely to be diagnosed in youth of the socially difficult, the versatile personality problems, and the avoidant profiles. Specifically, Cluster A diagnoses were most common in the socially difficult profile, Cluster C diagnoses in the avoidant profile, and others in the versatile personality problems profile. Personality disorder diagnoses were rarely present among youth of the baseline and the goal-oriented profiles. The versatile personality problems profile showed the highest rate of DSM clinical disorders. Differences among profiles were specifically found, e.g., for externalizing disorders (ADHD and conduct disorders most prevalent in the socially difficult profile) and internalizing disorders (affective and anxiety disorders most prevalent in the versatile personality problems and avoidant profiles). Any criminal behavior before assessment was most common in the versatile personality problems profile, whereas prior non-violent delinquency was most prevalent in the socially difficult profile. No differences emerged concerning violent delinquency before assessment. Future general and non-violent crime (after assessment) was most common in youth of the socially difficult profile. The avoidant profile showed the lowest crime rates. Regarding covariates, male adolescents were overrepresented in the socially difficult profile and underrepresented in the avoidant profile. Whereas the indifferent profile had the highest proportion of youth of Swiss nationality and low SES; Swiss youths were underrepresented in the goal-oriented profile.

### 3.4. Predictive Effects of ACEs and Personality Profiles on Future Crime

In order to examine predictive effects of ACEs and personality profiles on future general, violent, and non-violent delinquency, we performed several binary logistic regression analyses (see Table 3). First, we calculated univariate models with the ETI sum score (Model 1a) and the distinct LPA personality profiles (Model 1b; the baseline profile served as reference group). Second, we considered both ETI sum scores and personality profiles conjointly (Model 2). Third, we added prior general delinquency and the covariates age, sex, Swiss nationality, and low SES (Model 3). The ETI sum score did not predict future delinquency, in neither univariate nor multivariate models. Affiliation to the avoidant profile predicted desistance from future general and non-violent crime, whereas youth from the socially difficult profile were more likely to get involved in future general and non-violent crime even under control of the effects of ACEs, prior delinquency, and sociodemographic covariates. Prior delinquency and male sex increased the likelihood of future general, violent, and non-violent delinquency. No other predictors emerged for future violent crime involvement.

### 3.5. Predictive Effects of ACEs and Dimensional Personality Variables on Future Crime

In addition, we conducted equivalent analyses with the dimensional personality variables (see Table 4). First, we calculated univariate models with the ETI sum score (Model 1a) and each dimensional personality variable (Model 1b). Second, we considered both ETI sum scores and all dimensional personality variables conjointly (Model 2). Third, we added prior general delinquency and the covariates age, sex, Swiss nationality, and low SES (Model 3). Again, the ETI sum score did not predict future delinquency. In univariate analyses, the YPI sum score as well as the JTCI novelty seeking and SCID-II Cluster B scores were positively related to future general, violent, and non-violent crime, whereas SCID-II Cluster C scores were negatively associated with future general and non-violent crime. However, when all dimensional personality variables and ACEs were conjointly considered under control of the effects of prior delinquency and sociodemographic covariates (Model 3), only the positive predictive effects of SCID-II Cluster B scores on future general and violent offending and the negative predictive effects of SCID-II Cluster C scores on future general and non-violent offending remained significant. Again, prior delinquency and male sex increased the likelihood of future general, violent, and non-violent delinquency.

## 4. Discussion

The present study extends the knowledge on the associations between ACEs, juvenile personality, and crime by investigating the respective effects in a relatively large, heterogeneous high-risk sample of male and female adolescents living in child-welfare or residential care/juvenile justice institutions. By implementing a comprehensive approach considering cumulative ACEs and personality profiles instead of single ACEs and personality traits alone, the current results may inspire future research as well as prevention and intervention practice aiming at reducing the risk of (repeated) juvenile crime involvement. 

### 4.1. Prevalence of ACEs, Personality Traits, Psychopathologcial Disturbances, and Crime

As expected, we found high rates of ACEs in the present sample, with more than 82% of the juveniles reporting at least one, and more than half of them reporting at least three different ACEs. Prevalence rates in the present sample were comparable to and even slightly exceeded rates found in delinquent and non-delinquent institutionalized youth in previous studies (e.g., [5,28]). This might be because our sample was somewhat more heterogeneous, e.g., with respect to crime involvement (about one third of the juveniles had been convicted before and one third after the assessment) than samples in previous studies, and that measures of ACEs differed among studies. Personality disturbances in terms of categorical personality disorders were found in one fifth of the sample. Prevalence of psychopathology was also high in the present sample with more than 82% showing at least one psychiatric diagnosis, most frequently conduct disorders, affective disorders, and ADHD. Both findings underline previous research pointing to high rates of maladaptive personality traits and psychopathology among high-risk youth (e.g., [5,21,22]). Thus, our findings emphasize that high-risk, institutionalized youth display a highly burdened population with respect to ACEs, personality-related, and psychopathological disturbances. 

### 4.2. Personality Profiles

The current findings suggest that valid profiles can be derived based on the simultaneous consideration of non-pathological and pathological personality features. Previous studies have identified personality profiles based on psychopathy measures only (e.g., [37,58]), but a more holistic approach including different features of personality (including psychopathic traits, temperament, and measures of personality disorders) may lead to a more profound understanding of youth burdened by ACEs and at risk of (future) delinquency. Based on dimensional, self-reported measures of psychopathy, temperament, and personality disorders, we did not only find two profiles representing high and low personality-related disturbances, but empirically derived six distinct personality profiles among our high-risk youth sample. Although a great proportion of youth showed rather inconspicuous personality traits, most of the sample could be assigned to divergent personality profiles.

#### 4.2.1. Baseline Profile

About 42.1% of the sample were assigned to the baseline profile with rather inconspicuous values across all assessed personality variables. Males were overrepresented in this profile compared to females. Juveniles of the baseline profile were seldom burdened with multiple ACEs, clinician-administered personality disorders, and affective disorders. However, almost half of the adolescents with previous and future delinquency belonged to the baseline profile. These youths may represent juveniles with an occurrence of criminal behavior that is rather typical for adolescence but unrelated to ACEs, personality, or psychopathology [1].

#### 4.2.2. Socially Difficult Profile

With comparatively high scores on psychopathy, novelty seeking, and SCID-II Cluster B traits, 10.8% of the sample were assigned to the socially difficult personality profile. Rates of ACEs were high, and clinical personality disorders (especially Cluster A) and externalizing psychiatric diagnoses (i.e., conduct disorders, ADHD) were more common in this profile than in others, whereas sex distribution was balanced. Moreover, although not overrepresented among youth with prior delinquency, juveniles of the socially difficult profile showed high rates of future (non-violent) crime. Class assignment to the socially difficult profile remained a significant predictor of future general and non-violent delinquency in multivariate models. The socially difficult personality profile found in the present study appears comparable to the criminal personality profile and the personality traits associated with delinquency in the study by Krischer et al. [22], with high scores on conduct problems (i.e., conduct disorders), sensation seeking (i.e., novelty seeking), affective lability (i.e., SCID-II Cluster B traits), impulsivity (i.e., ADHD), and callousness (i.e., psychopathy). 

#### 4.2.3. Versatile Personality Problems Profile

A small proportion of the sample (6.7%) was assigned to the versatile personality problems profile, which showed high levels on all dimensional SCID-II personality disorder scales. Juveniles from this profile appeared to be most burdened with high ACE rates and DSM clinical diagnoses (especially affective disorders). Also, clinician-administered DSM-IV personality disorders were most commonly diagnosed in this profile, especially with regard to unspecified/combined personality disorders. Although the proportion of juveniles from this profile was relatively high among those who had been convicted for any previous crime, assignment to the versatile personality problems profile was not predictive of future crime involvement. Thus, juveniles from this profile may represent a highly burdened subgroup among institutionalized youth with significant need for treatment, which, however, may rather focus on clinical than forensic (crime-related) aspects.

#### 4.2.4. Avoidant Profile

With high values on dimensional SCID-II Cluster C ratings but rather inconspicuous scores on other personality traits, 14.6% of the sample were assigned to the avoidant personality profile. Females and youth of Swiss nationality were overrepresented in this profile. Clinician-administered DSM Cluster C personality disorders were comparatively common, whereas psychiatric disorders in terms of substance use disorders and conduct disorders were relatively rare. The number of ACEs did not stand out compared to the total sample. Most strikingly, juveniles from the avoidant personality profile were clearly underrepresented among those with prior and future delinquent behaviors; moreover, assignment to the avoidant profile had a protective effect regarding future offending even in multivariate models. These findings are in line with previous research stating that Cluster C personality disorders were not related to increased risk of future crime [11]. This may be since youth with avoidant personality traits could rather avoid or step away from situations that elicit the risk of criminal conduct or violent escalation.

#### 4.2.5. Goal-Oriented Profile

Juveniles with rather inconspicuous scores on maladaptive personality traits but high values on reward dependence and persistence were assigned to the goal-oriented personality profile (14.3%). Compared to other profiles, adolescents without Swiss nationality and low SES were overrepresented in this profile. Goal-oriented youth were seldomly burdened by ACEs, clinician-administered personality disorders, or psychiatric disorders, and showed no specifics in terms of prior or future delinquency. This finding contributes to research assuming that crime may rather be committed by burdened adolescents (e.g., [8]). Moreover, although reward dependence per se may reflect a rather ambiguous trait as it may also enhance the risk of criminal conduct (e.g., [59]), a pattern of high reward dependence and high persistence may, conversely, be rather functional for goal achievement and, thus, prevent frustration and subsequent crime.

#### 4.2.6. Indifferent Profile

Finally, about 11.4% of the sample showing inconspicuous scores on clinical personality scales and low novelty seeking, reward dependence, and persistence were assigned to the indifferent personality profile. Juveniles of this profile were comparatively often from families with low SES and Swiss nationality. No specific patterns emerged concerning ACEs, clinician-administered personality disorders, psychiatric disorders, and delinquency. Thus, juveniles of this profile showed low psychosocial burden despite coming from low SES backgrounds. It may be that the indifferent personality traits led juveniles to rather engage in resignation regarding maladaptive outcomes. Equally, although often discussed in relation to crime, low SES may not enhance the risk of criminal behavior per se but rather in combination with other risk factors (e.g., [60]).

### 4.3. Prediction of Future Crime by ACEs and Personality 

Our data suggests that ACEs had no effect on risk of future crime, neither in univariate nor multivariate models. This result contradicts previous findings that emphasized the role of ACEs in the prediction of repeated juvenile crime involvement (e.g., [61]), yet contributes to research that did not find respective associations [62]. We might have failed to detect previously mentioned predictive effects due to several reasons. First, as mentioned above, our measure of ACEs was somewhat different and broader than measures used in previous studies. Second, the overall high prevalence rates of ACEs in the current high-risk sample of juveniles with and without previous offenses may have affected the results. Third, although serving as convenient way to consider the co-existence of multiple types of ACEs, a cumulative ACE score has also been criticized because it cannot account for the effects of specific patterns of ACEs (e.g., [4,63]). Fourth, compared to studies that emphasized the ACE-delinquency link in juvenile justice samples, only about one third of our high-risk sample had been convicted of criminal behavior before and after the assessment. Thus, future delinquency in the present study was not entirely equivalent to re-offending reported in studies on criminal youth but also included future first time offending. Fifth, the sole reliance on official crime data (convictions) may have led to potential underreporting of crime. Furthermore, delinquency may only be one way to deal with psychosocial burden. Despite the elevated ACE prevalence in our high-risk sample, a vast proportion of the juveniles examined in the present study may have been affected differently by ACEs, e.g., holding a higher risk of developing mental disturbances rather than engaging in future crime. 

Regarding youth personality, two profiles turned out to be of major relevance in the prediction of future delinquency. On the one hand, avoidant personality traits appeared to be protective of crime involvement in general, whereas socially difficult personality traits increased the risk of (future) general and non-violent delinquency, even when controlled for ACEs, sociodemographic covariates, and prior delinquency. Thus, juveniles with patterns of socially difficult personality features (i.e., psychopathy, novelty seeking, and SCID-II Cluster B traits) display a subgroup at specific risk of future delinquency among the high-risk sample of institutionalized youth. Interestingly, when considered by variable-centered analyses, psychopathic features and novelty seeking, were, in contrast to SCID-II Cluster B and C traits, only associated with future delinquency in univariate, but not multivariate analyses. This finding is contrary to previous research indicating that temperament was more strongly associated with delinquency than ACEs and psychopathic traits [27]. Similar to criticism regarding the assessment of ACEs (e.g., [4]), research on adolescent personality may also benefit from considering empirically derived profiles in addition to variable-centered approaches. 

Apart from ACEs and personality, male sex and prior delinquency proved to be consistent predictors of future crime involvement, contributing to previous research (e.g., [27]) and implementation, as especially male juveniles and those who have been criminally involved in their pasts need special attention regarding prevention and treatment.

### 4.4. Strengths and Limitations

The present study offers several considerable strengths but also some qualifications that need to be considered when interpreting our results and extracting implications. First, we were able to examine a high-risk sample of juveniles living in child welfare and juvenile justice institutions. The sample consisted of both male and female juveniles as well as previously delinquent and non-delinquent youth. Both self-rating and clinician-administered data were assessed, and we derived crime data from official state databases covering a follow-up period up to 10 years. By implementing LPA, personality profiles were empirically defined by bottom-up instead of theoretical top-down approaches. However, self-ratings as well as clinician-administered ratings are not without subjective bias, and the sole reliance on official crime data (convictions) may have led to potential underreporting of crime. Moreover, future research may gain deeper insights into the given topic by implementing mixed-methods studies that consider both quantitative and qualitative data. The number of juveniles in some of the personality profiles was rather small, reducing statistical power, limiting generalizability to other (especially community) youth samples, and, moreover, impeding the investigation of further relevant aspects such as sex differences. As mentioned above, considering ACEs by a cumulative score may have disguised more subtle effects of distinct ACE patterns; yet, examining the single and shared impact of unique ACEs and specified ACE patterns was beyond the scope of the present study. Additionally, we were not able to further investigate the potential effects of psychiatric diagnoses, which were quite common in our sample, although externalizing disorders, in particular, were found to enhance the risk of (repeated) criminal conduct (e.g., [64,65]). Furthermore, it is not clear whether psychiatric disturbances were present before placement or have developed during placement. Finally, some more restrictions related to the design of the underlying MAZ. study apply, too (e.g., regarding placement trajectory; see [40]). 

## 5. Conclusions

Based on data from a relatively large, heterogeneous high-risk sample of male and female adolescents living in child-welfare and residential care/juvenile justice institutions, we found that cumulative ACEs did not predict future crime involvement. However, distinct personality profiles emerged based on measures of psychopathy, temperament, and personality disorders, which differed regarding ACEs, personality disturbances, clinical psychopathology, and future delinquency. A socially difficult personality profile was associated with increased risk of future crime, whereas avoidant personality traits appeared rather protective. Findings indicate that the role of ACEs in the prediction of juvenile delinquency is still not sufficiently clear and that relying on single personality traits alone may be insufficient in the explanation of juvenile crime. A comprehensive but individualized consideration of ACEs, youth personality, psychiatric disturbances, and delinquent risk is needed in both research and clinical practice in order to derive and implement promising prevention and intervention approaches that meet a juvenile’s individual needs and reduce adolescents’ psychosocial burden and risk of future crime perpetration.

## Figures and Tables

**Table 2:** Main variables of interest in the six distinct personality profiles.

Variables of Interest	Baseline (*n* = 144)		Socially Difficult (*n* = 37)		Versatile Personality Problems (*n* = 23)		Avoidant (*n* = 50)		Goal Oriented (*n* = 49)		Indifferent (*n* = 39)		
	***M* (*SD*)**		***M* (*SD*)**		***M* (*SD*)**		***M* (*SD*)**		***M* (*SD*)**		***M* (*SD*)**		***F* (5, 336)**
**ACEs**													
ETI sum	2.73 ^a^ (2.52)		4.00 ^a,b^ (2.21)		5.78 ^b^ (2.47)		3.32 ^a^ (2.44)		2.65 ^a^ (2.54)		3.64 ^a^ (2.76)		7.36 ***
**Personality (dim.)**													
YPI sum	115.31 ^a^ (19.40)		129.73 ^b^ (21.01)		116.43 ^a^ (20.74)		98.44^c^ (18.64)		86.27 ^c^ (13.85)		119.41 ^a^ (16.23)		33.16 ***
JTCI novelty seeking	33.34 ^a^ (7.76)		36.27 ^a^ (8.96)		29.74 ^a^ (8.44)		29.26 ^a^ (8.77)		22.10 ^b^ (6.22)		20.38 ^b^ (9.28)		33.33 ***
JTCI harm avoidance	24.69 ^a^ (8.20)		23.43 ^a,b^ (7.69)		26.74 ^a^ (6.78)		25.84 ^a^ (8.30)		18.59 ^b^ (9.00)		21.26 ^a,b^ (7.37)		6.37 ***
JTCI reward dependence	38.68 ^a^ (8.54)		38.97 ^a,b,c^ (6.81)		32.96 ^b^ (7.75)		35.78 ^a,b^ (9.42)		44.16 ^c^ (8.33)		25.18 ^d^ (5.02)		27.28 ***
JTCI persistence	29.42 ^a^ (7.12)		29.08 ^a^ (6.29)		27.22 ^a,b^ (5.08)		28.92 ^a^ (8.66)		42.41 ^c^ (6.26)		21.92 ^b^ (5.65)		43.65 ***
SCID-II Cluster A (dim.)	24.46 ^a^ (2.64)		31.43 ^b^ (4.62)		44.30 ^c^ (5.11)		28.70 ^b,d^ (4.50)		25.90 ^a^ (3.47)		26.28 ^a,d^ (3.78)		130.65 ***
SCID-II Cluster B (dim.)	38.78 ^a^ (5.93)		56.68 ^b^ (9.89)		57.65 ^b^ (11.04)		40.74 ^a^ (7.47)		38.63 ^a^ (5.44)		41.77 ^a^ (7.17)		60.53 ***
SCID-II Cluster C (dim.)	25.07 ^a^ (2.92)		29.00 ^b^ (4.23)		36.48 ^c^ (4.00)		38.84 ^c^ (4.33)		27.18 ^b^ (3.29)		26.38 ^a,b^ (3.15)		146.06 ***
SCID-II other (dim.)	15.64 ^a^ (2.05)		22.32 ^b^ (3.98)		26.39 ^c^ (4.27)		21.86 ^b^ (3.94)		16.57 ^a^ (3.18)		16.59 ^a^ (2.89)		86.08 ***
**Psychopathology**													
K-SADS-PL sum	1.60 (1.20) ^a^		2.22 (1.11) ^a,b^		2.70 (1.69) ^b^		1.74 (1.23) ^a^		1.57 (1.06) ^a^		1.67 (1.61) ^a^		4.16 ***
	***n* (%)**	***AR***	***n* (%)**	***AR***	***n* (%)**	***AR***	***n* (%)**	***AR***	***n* (%)**	***AR***	***n* (%)**	***AR***	**χ^2^(5)**
**Personality disorders (cat.)**													
SCID-II no PD	134 (49.5)	5.5	21 (7.8)	−3.5	5 (1.5)	−7.0	30 (11.1)	−3.6	47 (17.4)	3.1	33 (12.2)	0.9	93.54 ***
SCID-II Cluster A (cat.)	1 (16.7)	−1.3	4 (66.7)	4.4	0 (0.0)	−0.7	1 (16.7)	0.1	0 (0.0)	−1.0	0 (0.0)	−0.9	20.55 ***
SCID-II Cluster B (cat.)	7 (41.2)	−0.1	4 (23.5)	1.7	1 (5.9)	−0.1	1 (5.9)	−1.0	1 (5.9)	−1.0	3 (17.6)	0.8	5.13
SCID-II Cluster C (cat.)	0 (0.0)	−2.6	0 (0.0)	−1.1	0 (0.0)	−0.8	9 (100.0)	7.3	0 (0.0)	−1.2	0 (0.0)	−1.1	53.98 ***
SCID-II other (cat.)	2 (5.0)	−5.1	8 (20.0)	2.0	17 (42.5)	9.6	9 (22.5)	1.5	1 (2.5)	−2.3	3 (7.5)	−0.8	111.50 ***
**Prior delinquency**													
Any	55 (49.5)	1.9	14 (12.6)	0.7	13 (11.7)	2.6	3 (2.7)	−4.3	12 (10.8)	−1.3	14 (12.6 )	0.5	26.32 ***
Violent	9 (34.6)	−0.8	4 (15.4)	0.8	4 (15.4)	1.8	1 (3.8)	−1.6	3 (11.5)	−0.4	5 (19.2)	1.3	7.95
Non-Violent	55 (52.4)	2.6	14 (13.3)	1.0	11 (10.5)	1.8	2 (1.9)	−4.4	12 (11.4)	−1.0	11 (10.5)	−0.4	25.61 ***
**Future delinquency**													
Any	53 (48.2)	1.6	20 (18.2)	3.0	9 (8.2)	0.7	4 (3.6)	−4.0	12 (10.9)	−1.2	12 (10.9)	−0.2	24.80 ***
Violent	20 (54.1)	1.6	7 (18.9)	1.7	4 (10.8)	1.1	0 (0.0)	−2.7	3 (8.1)	−1.1	3 (8.1)	−0.7	12.53 *
Non-Violent	48 (47.5)	1.3	19 (18.8)	3.1	8 (7.9)	0.6	4 (4.0)	−3.6	10 (9.9)	−1.5	12 (11.9)	0.2	22.90 ***

Note. *N* = 342. dim = dimensional, cat = categorical, PD = personality disorder. AR adjusted residuals. Significant deviations from expected distribution with AR ≤ − 2.0 or AR ≥ 2.0. Groups with the same subscripts (a, b, c, and d) did not significantly differ from each other. * *p* ≤ 0.05, *** *p* ≤ 0.001.

**Table 3:** Binary logistic regression analyses on future delinquency with ACEs, personality profiles, and covariates.

Model	Independent Variables	Future Delinquency					
		Any		Violent		Non-Violent	
		OR	95%–CI	OR	95%–CI	OR	95%–CI
Model 1a	ETI sum	0.96	0.88–1.05	0.99	0.86–1.12	0.99	0.91–1.08
Model 1b	Socially Difficult	2.00	0.94–4.23	1.43	0.55–3.70	2.09	0.99–4.45
	Versatile Personality Problems	1.16	0.46–2.92	1.27	0.39–4.14	1.13	0.44–2.89
	Avoidant	0.08 ***	0.02–0.34	0.00	0.00–0.00	0.09 ***	0.02–0.40
	Goal Oriented	0.58	0.27–1.21	0.39	0.11–1.37	0.54	0.24–1.17
	Indifferent	0.77	0.35–1.71	0.35	0.08-1.56	0.91	0.41–2.02
Model 2	Socially Difficult	2.15 *	1.00–4.62	1.46	0.55–3.84	2.15 *	1.00–4.62
	Versatile Personality Problems	1.38	0.53–3.64	1.33	0.38–4.67	1.21	0.45–3.23
	Avoidant	0.08 ***	0.02–0.35	0.00	0.00–0.00	0.09 ***	0.02–0.40
	Goal Oriented	0.57	0.27–1.20	0.39	0.11–1.37	0.53	0.24–1.17
	Indifferent	0.82	0.37–1.84	0.35	0.08–1.60	0.93	0.42–2.08
	ETI sum	0.94	0.85–1.04	0.98	0.85–1.14	0.98	0.88–1.08
Model 3	Socially Difficult	2.40 *	1.02–5.62	1.48	0.54–4.06	2.45 *	1.04–5.74
	Versatile Personality Problems	1.20	0.41–3.50	1.31	0.35–4.87	1.05	0.35–3.11
	Avoidant	0.16 *	0.04–0.70	0.00	0.00–0.00	0.19 *	0.04–0.84
	Goal Oriented	0.65	0.28–1.051	0.43	0.11–1.62	0.60	0.25–1.45
	Indifferent	1.00	0.41–2.45	0.41	0.09–1.93	1.24	0.50–3.06
	ETI sum	0.95	0.84–1.06	0.99	0.84–1.15	0.99	0.88–1.11
	Prior general delinquency	3.63 ***	1.98–6.67	2.79 **	1.22–6.38	3.50 ***	1.89–6.48
	Age	0.90	0.75–1.08	0.83	0.64–1.08	0.90	0.75–1.09
	Sex (males = 0, females = 1)	0.22 ***	0.11–0.45	0.33 *	0.11–0.98	0.21***	0.10–0.43
	Swiss nationality	0.86	0.92–1.92	0.80	0.29–2.19	0.72	0.86–0.38
	Low SES	0.72	0.38–1.40	0.80	0.33–1.98	0.63	0.32–1.25

Note. Reference group: Baseline profile. OR = odds ratio; CI = confidence interval. * *p* ≤ 0.05, ** *p* ≤ 0.01, *** *p* ≤ 0.001.

**Table 4:** Binary logistic regression analyses on future delinquency with ACEs, dimensional personality variables, and covariates.

Model	Independent Variables	Future Delinquency					
		Any		Violent		Non-Violent	
		OR	95%–CI	OR	95%–CI	OR	95%–CI
Model 1a	ETI sum	0.96	0.88–1.05	0.99	0.86-1.12	0.99	0.91–1.08
Model 1b	YPI sum	1.03 ***	1.02–1.04	1.03 ***	1.02–1.05	1.02 ***	0.01–1.04
	JTCI novelty seeking	1.03 **	1.01–1.06	1.05 **	1.01–1.09	1.03 *	1.01–1.06
	JTCI harm avoidance	0.99	0.96–1.01	1.00	0.96–1.04	0.98	0.95–1.01
	JTCI reward dependence	0.99	0.97–1.01	1.00	0.97–1.04	0.98	0.96–1.01
	JTCI persistence	0.99	0.97–1.02	0.98	0.94–1.02	0.98	0.96–1.01
	SCID-II Cluster A	1.02	0.98–1.06	1.02	0.97–1.08	1.02	0.99–1.06
	SCID-II Cluster B	1.03 **	1.01–1.06	1.04**	1.01–1.07	1.03 *	1.01–1.05
	SCID-II Cluster C	0.93 ***	0.89–0.97	0.95	0.89–1.01	0.93 ***	0.89–0.97
	SCID-II other	0.98	0.94–1.03	0.98	0.90–1.06	0.98	0.93–1.04
Model 2	YPI sum	1.02 **	1.01–1.03	1.02 *	1.00–1.04	1.02 *	1.00–1.03
	JTCI novelty seeking	1.02	0.99–1.05	1.04	1.00–1.09	1.03	0.99–1.06
	JTCI harm avoidance	0.99	0.96–1.02	1.01	0.97–1.06	0.99	0.95–1.02
	JTCI reward dependence	0.99	0.96–1.02	1.01	0.97–1.06	0.98	0.95–1.01
	JTCI persistence	1.01	0.97–1.04	0.99	0.94–1.04	1.00	0.97–1.04
	SCID-II Cluster A	1.07 *	1.01–1.13	1.05	0.97–1.14	1.08 *	1.02–1.14
	SCID-II Cluster B	1.04	1.00–1.07	1.04	0.99–1.08	1.02	0.99–1.06
	SCID-II Cluster C	0.89 ***	0.83–0.95	0.94	0.86–1.04	0.89 ***	0.83–0.95
	SCID-II other	0.98	0.90–1.06	0.94	0.84–1.06	0.98	0.90–1.06
	ETI sum	0.90	0.80–1.00	0.91	0.77–1.08	0.94	0.84–1.05
Model 3	YPI sum	1.01	1.00–1.03	1.02	1.00–1.04	1.01	0.99–1.02
	JTCI novelty seeking	1.02	0.99–1.06	1.04	1.00–1.09	1.03	0.99–1.06
	JTCI harm avoidance	1.00	0.97–1.04	1.02	0.97–1.07	1.00	0.96–1.03
	JTCI reward dependence	0.99	0.95–1.02	1.02	0.97–1.07	0.98	0.94–1.01
	JTCI persistence	1.00	0.97–1.04	0.99	0.94–1.04	1.00	0.96–1.04
	SCID-II Cluster A	1.02	0.95–1.08	1.03	0.94–1.12	1.03	0.96–1.09
	SCID-II Cluster B	1.05 *	1.01–1.09	1.05	1.00–1.10	1.04	1.00–1.08
	SCID-II Cluster C	0.90 **	0.84–0.97	0.97	0.88–1.07	0.90 **	0.84–0.97
	SCID-II other	1.00	0.92–1.10	0.93	0.82–1.06	1.00	0.91–1.10
	ETI sum	0.91	0.81–1.03	0.93	0.79–1.11	0.96	0.85–1.08
	Prior Delinquency	3.52 ***	1.90–6.54	3.06 *	1.29–7.23	3.26 ***	1.74–6.08
	Age	0.88	0.72–1.06	0.73 *	0.54–0.99	0.89	0.73–1.09
	Sex (males = 0, females = 1)	0.21***	0.10–0.45	0.28 *	0.08–0.93	0.20 ***	0.09–0.44
	Nationality	0.81	0.35–1.88	0.61	0.21–1.79	0.79	0.33–1.85
	Low SES	0.77	0.39–1.52	0.87	0.34–2.23	0.66	0.33–1.32

Note. OR = odds ratio; CI = confidence interval.* *p* ≤ 0.05, ** *p* ≤ 0.01, *** *p* ≤ 0.001.

## Data Availability

The data presented in this study are available on request from the last author (C.B.).

## Appendix A

**Table A1:** Distribution of variables of interest in the total sample and the six personality profiles.

	Total Sample (*N* = 342)	Baseline (*n* = 144)		Socially Difficult (*n* = 37)		Versatile Personality Problems (*n* = 23)		Avoidant (*n* = 50)		Goal Oriented (*n* = 49)		Indifferent (*n* = 39)		
**ACEs**	***M* (*SD*)**	***M* (*SD*)**		***M* (*SD*)**		***M* (*SD*)**		***M* (*SD*)**		***M* (*SD*)**		***M* (*SD*)**		***F* (5, 336)**
ETI sum	3.25 (2.62)	2.73 ^a^ (2.52)		4.00 ^a,b^ (2.21)		5.78 ^b^ (2.47)		3.32 ^a^ (2.44)		2.65 ^a^ (2.54)		3.64 ^a^ (2.76)		7.36 ***
	***n* (%)**	***n* (%)**	***AR***	***n* (%)**	***AR***	***n* (%)**	***AR***	***n* (%)**	***AR***	***n* (%)**	***AR***	***n* (%)**	***AR***	**χ^2^ (5)**
ETI sum = 0	61 (17.8)	36 (59.0)	3.0	1 (1.6)	−2.5	0 (0.0)	−2.3	6 (9.8)	−1.2	9 (14.8)	0.1	9 (14.8)	0.9	17.72 **
ETI sum = 1	47 (13.7)	19 (40.4)	−0.3	6 (12.8)	0.5	0 (0.0)	−2.0	7 (14.9)	0.1	14 (29.8)	3.3	1 (2.1)	−2.2	17.10 **
ETI sum = 2	40 (11.7)	18 (45.0)	0.4	4 (10.0)	−0.2	2 (5.0)	−0.5	9 (22.5)	1.5	5 (12.5)	−0.4	2 (5.0)	−1.4	3.98
ETI sum ≥ 3	194 (56.8)	71 (36.6)	-2.4	26 (13.4)	1.8	21 (10.8)	3.5	28 (14.4)	−0.1	21 (10.8)	−2.1	27 (13.9)	1.7	23.53 ***
**Personality**	***M* (*SD*)**	***M* (*SD*)**		***M* (*SD*)**		***M* (*SD*)**		***M* (*SD*)**		***M* (*SD*)**		***M* (*SD*)**		***F* (5, 336)**
YPI sum	110.78 (22.48)	115.31 ^a^ (19.40)		129.73 ^b^ (21.01)		116.43 ^a^ (20.74)		98.44^c^ (18.64)		86.27 ^c^ (13.85)		119.41 ^a^ (16.23)		33.16 ***
JTCI novelty seeking	29.73 (9.28)	33.34 ^a^ (7.76)		36.27 ^a^ (8.96)		29.74 ^a^ (8.44)		29.26 ^a^ (8.77)		22.10 ^b^ (6.22)		20.38 ^b^ (9.28)		33.33 ***
JTCI harm avoidance	23.59 (8.42)	24.69 ^a^ (8.20)		23.43 ^a,b^ (7.69)		26.74 ^a^ (6.78)		25.84 ^a^ (8.30)		18.59 ^b^ (9.00)		21.26 ^a,b^ (7.37)		6.37 ***
JTCI reward dependence	37.15 (9.54)	38.68 ^a^ (8.54)		38.97 ^a,b,c^ (6.81)		32.96 ^b^ (7.75)		35.78 ^a,b^ (9.42)		44.16 ^c^ (8.33)		25.18 ^d^ (5.02)		27.28 ***
JTCI persistence	30.17 (8.80)	29.42 ^a^ (7.12)		29.08 ^a^ (6.29)		27.22 ^a,b^ (5.08)		28.92 ^a^ (8.66)		42.41 ^c^ (6.26)		21.92 ^b^ (5.65)		43.65 ***
SCID-II Cluster A (dim.)	27.58 (6.20)	24.46 ^a^ (2.64)		31.43 ^b^ (4.62)		44.30 ^c^ (5.11)		28.70 ^b,d^ (4.50)		25.90 ^a^ (3.47)		26.28 ^a,d^ (3.78)		130.65 ***
SCID-II Cluster B (dim.)	42.59 (6.12)	38.78 ^a^ (5.93)		56.68 ^b^ (9.89)		57.65 ^b^ (11.04)		40.74 ^a^ (7.47)		38.63 ^a^ (5.44)		41.77 ^a^ (7.17)		60.53 ***
SCID-II Cluster C (dim.)	28.73 (6.12)	25.07 ^a^ (2.92)		29.00 ^b^ (4.23)		36.48 ^c^ (4.00)		38.84 ^c^ (4.33)		27.18 ^b^ (3.29)		26.38 ^a,b^ (3.15)		146.06 ***
SCID-II other (dim.)	18.24 (4.58)	15.64 ^a^ (2.05)		22.32 ^b^ (3.98)		26.39 ^c^ (4.27)		21.86 ^b^ (3.94)		16.57 ^a^ (3.18)		16.59 ^a^ (2.89)		86.08 ***
	***n* (%)**	***n* (%)**	***AR***	***n* (%)**	***AR***	***n* (%)**	***AR***	***n* (%)**	***AR***	***n* (%)**	***AR***	***n* (%)**	***AR***	**χ^2^ (5)**
SCID-II no PD	270 (78.9)	134 (49.5)	5.5	21 (7.8)	−3.5	5 (1.5)	−7.0	30 (11.1)	−3.6	47 (17.4)	3.1	33 (12.2)	0.9	93.54 ***
SCID-II Cluster A (cat.)	6 (1.8)	1 (16.7)	−1.3	4 (66.7)	4.4	0 (0.0)	−0.7	1 (16.7)	0.1	0 (0.0)	−1.0	0 (0.0)	−0.09	20.55 ***
SCID-II Cluster B (cat.)	17 (5.9)	7 (41.2)	−0.1	4 (23.5)	1.7	1 (5.9)	−0.1	1 (5.9)	−1.0	1 (5.9)	−1.0	3 (17.6)	0.8	5.13
SCID-II Cluster C (cat.)	9 (2.6)	0 (0.0)	−2.6	0 (0.0)	−1.1	0 (0.0)	−0.8	9 (100.0)	7.3	0 (0.0)	−1.2	0 (0.0)	−1.1	53.98 ***
SCID-II other (cat.)	40 (11.7)	2 (5.0)	−5.1	8 (20.0)	2.0	17 (42.5)	9.6	9 (22.5)	1.5	1 (2.5)	−2.3	3 (7.5)	−0.8	111.50 ***
**Psychopathology**	***M* (*SD*)**	***M* (*SD*)**		***M* (*SD*)**		***M* (*SD*)**		***M* (*SD*)**		***M* (*SD*)**		***M* (*SD*)**		***F* (5, 336)**
K-SADS-PL sum	1.77 (1.30)	1.60 (1.20) ^a^		2.22 (1.11) ^a,b^		2.70 (1.69) ^b^		1.74 (1.23) ^a^		1.57 (1.06) ^a^		1.67 (1.61) ^a^		4.16***
	***n* (%)**	***n* (%)**	***AR***	***n* (%)**	***AR***	***n* (%)**	***AR***	***n* (%)**	***AR***	***n* (%)**	***AR***	***n* (%)**	***AR***	**χ^2^ (5)**
K-SADS-PL sum = 0	60 (17.3)	28 (46.7)	0.8	2 (3.3)	−2.1	2 (3.3)	−1.2	7 (11.7)	−0.7	9 (15.0)	0.2	12 (20.0)	2.3	10.55
K-SADS-PL sum = 1	92 (26.9)	41 (0.6)	0.6	7 (7.6	−1.2	4 (4.3)	−1.1	18 (19.6)	1.6	13 (14.1)	−0.1	9 (9.8)	−0.6	4.84
K-SADS-PL sum = 2	105 (30.7)	48 (45.7)	0.9	14 (13.3)	1.0	6 (5.7)	−0.5	12 (11.4)	−1.1	19 (18.1)	1.3	6 (5.7)	−2.2	8.44
K-SADS-PL sum ≥ 3	85 (25.1)	27 (31.8)	-2.2	14 (16.5)	1.9	11 (12.9)	2.6	13 (15.3)	0.2	8 (9.4)	−1.5	12 (14.1)	0.9	15.39 **
K-SADS-PL substance use	98 (28.7)	48 (49.0)	1.6	13 (14.3)	1.3	11 (11.2)	2.1	5 (5.1)	−3.2	9 (9.2)	−1.8	11 (11.2)	0.1	18.31 **
K-SADS-PL schizophrenic	3 (0.9)	2 (66.7)	0.9	0 (0.0)	−0.6	1 (33.3)	1.8	0 (0.0)	−0.7	0 (0.0)	−0.7	0 (0.0)	−0.6	5.12
K-SADS-PL affective	105 (30.7)	28 (26.7)	−3.9	9 (8.6)	−0.9	15 (14.3)	3.7	24 (22.9)	2.8	19 (18.1)	1.3	10 (9.5)	−0.6	30.51***
K-SADS-PL anxiety	55 (16.1)	17 (30.9)	−1.8	4 (7.3)	−0.9	9 (16.4)	3.1	14 (25.5)	2.4	5 (9.1)	−1.2	6 (10.9)	0.0	18.06 **
K-SADS-PL obsessive-compulsive	7 (2.0)	1 (14.3)	−1.5	2 (28.6)	1.5	0 (0.0)	−0.7	1 (14.3)	0.0	2 (28.6)	1.1	1 (14.3)	0.3	4.91
K-SADS-PL traumatic	74 (21.6)	27 (36.5)	−1.1	10 (13.5)	0.8	9 (12.2)	2.1	13 (17.6)	0.8	8 (10.8)	−1.0	7 (9.5)	−0.5	6.91
K-SADS-PL dissociative	1 (0.3)	0 (0.0)	−0.9	0 (0.0)	−0.4	1 (100.0)	3.7	0 (0.0)	−0.4	0 (0.0)	−0.4	0 (0.0)	−0.4	13.78 *
K-SADS-PL somatic	1 (0.3)	0 (0.0)	−0.9	0 (0.0)	−0.4	0 (0.0)	−0.3	1 (100.0)	2.4	0 (0.0)	−0.4	0 (0.0)	−0.4	5.80
K-SADS-PL eating	4 (1.2)	1 (25.0)	−0.7	0 (0.0)	−0.7	0 (0.0)	−0.5	0 (0.0)	−0.8	2 (50.0)	2.0	1 (25.0)	0.9	5.87
K-SADS-PL ADHD	86 (25.1)	32 (37.2)	−1.1	19 (22.1)	3.8	4 (4.7)	−0.9	11 (12.8)	−0.6	13 (15.1)	0.2	7 (8.1)	−1.0	15.79 **
K-SADS-PL conduct	125 (36.5)	58 (46.4)	1.2	21 (16.8)	2.7	9 (7.2)	0.2	9 (7.2)	−3.0	12 (9.6)	−1.9	16 (12.8)	0.9	18.69 **
K-SADS-PL other	45 (13.2)	17 (37.8)	−0.6	3 (6.7)	−1.0	3 (6.7)	0.0	9 (20.0)	1.1	7 (15.6)	0.2	6 (13.3)	0.6	2.39
**Prior delinquency**	***n* (%)**	***n* (%)**	***AR***	***n* (%)**	***AR***	***n* (%)**	***AR***	***n* (%)**	***AR***	***n* (%)**	***AR***	***n* (%)**	***AR***	**χ^2^ (5)**
Any	111 (32.5)	55 (49.5)	1.9	14 (12.6)	0.7	13 (11.7)	2.6	3 (2.7)	−4.3	12 (10.8)	−1.3	14 (12.6)	0.5	26.32 ***
Violent	26 (7.6)	9 (34.6)	−0.8	4 (15.4)	0.8	4 (15.4)	1.8	1 (3.8)	−1.6	3 (11.5)	−0.4	5 (19.2)	1.3	7.95
Non-Violent	105 (30.7)	55 (52.4)	2.6	14 (13.3)	1.0	11 (10.5)	1.8	2 (1.9)	−4.4	12 (11.4)	−1.0	11 (10.5)	−0.4	25.61 ***
**Future delinquency**	***n* (%)**	***n* (%)**	***AR***	***n* (%)**	***AR***	***n* (%)**	***AR***	***n* (%)**	***AR***	***n* (%)**	***AR***	***n* (%)**	***AR***	**χ^2^ (5)**
Any	110 (32.2)	53 (48.2)	1.6	20 (18.2)	3.0	9 (8.2)	0.7	4 (3.6)	−4.0	12 (10.9)	−1.2	12 (10.9)	−0.2	24.80 ***
Violent	37 (10.8)	20 (54.1)	1.6	7 (18.9)	1.7	4 (10.8)	1.1	0 (0.0)	−2.7	3 (8.1)	−1.1	3 (8.1)	−0.7	12.53 *
Non-Violent	101 (29.5)	48 (47.5)	1.3	19 (18.8)	3.1	8 (7.9)	0.6	4 (4.0)	−3.6	10 (9.9)	−1.5	12 (11.9)	0.2	22.90 ***
**Sociodemographics**	***M* (*SD*)**	***M* (*SD*)**		***M* (*SD*)**		***M* (*SD*)**		***M* (*SD*)**		***M* (*SD*)**		***M* (*SD*)**		***F* (5, 99.56)**
Age	15.74 (1.61)	15.75 (1.46)		16.00 (1.55)		16.50 (1.73)		15.19 (1.97)		15.95 (1.74)		15.49 (1.74)		2.16
	***n* (%)**	***n* (%)**	***AR***	***n* (%)**	***AR***	***n* (%)**	***AR***	***n* (%)**	***AR***	***n* (%)**	***AR***	***n* (%)**	***AR***	**χ^2^ (5)**
Female	120 (35.1)	42 (35.0)	−2.0	9 (7.5)	−1.5	8 (6.7)	0.0	28 (23.3)	3.4	18 (15.0)	0.3	15 (12.5)	0.5	13.95 *
Male	222 (64.9)	102 (45.9)	2.0	28 (12.6)	1.5	15 (6.8)	0.0	22 (9.9)	−3.4	31 (14.0)	-0.3	24 (10.8)	−0.5	13.95 *
Swiss Nationality	293 (85.7)	125 (42.7)	0.5	29 (9.9)	−1.3	21 (7.2)	0.8	49 (16.7)	2.7	30 (10.2)	−5.3	39 (13.3)	2.7	38.92 ***
Low SES	79 (23.2)	30 (38.0)	−0.9	10 (12.7)	0.5	6 (7.6)	0.3	9 (11.4)	−0.8	6 (7.6)	−2.1	18 (22.8)	3.9	18.25 **

Note. *N* = 342. dim = dimensional, cat = categorical, PD = personality disorder. AR adjusted residuals. Significant deviations from expected distribution with AR ≤ −2.0 or AR ≥ 2.0. Groups with the same subscripts (a, b, c, d) did not significantly differ from each other. * *p* ≤ 0.05, ** *p* ≤ 0.01, *** *p* ≤ 0.001.
